# Breakpoint structure of the *Anopheles gambiae *2R*b *chromosomal inversion

**DOI:** 10.1186/1475-2875-9-293

**Published:** 2010-10-25

**Authors:** Neil F Lobo, Djibril M Sangaré, Allison A Regier, Kyanne R Reidenbach, David A Bretz, Maria V Sharakhova, Scott J Emrich, Sekou F Traore, Carlo Costantini, Nora J Besansky, Frank H Collins

**Affiliations:** 1 Eck Institute for Global Health, Department of Biological Sciences, University of Notre Dame, Notre Dame, IN 46556, USA; 2 Malaria Research and Training Center (MRTC), Bamako, Mali; 3 Department of Computer Science and Engineering, University of Notre Dame, Notre Dame, IN 46556, USA; 4 Institut de Recherche pour le Développement, Unité de Recherche R016, Montpellier, France; and Organisation de Coordination pour la Lutte contre les Endémies en Afrique Centrale, Yaounde, Cameroon; 5 Department of Entomology, Virginia Tech, Blacksburg, VA 24061, USA

## Abstract

**Background:**

Alternative arrangements of chromosome 2 inversions in *Anopheles gambiae *are important sources of population structure, and are associated with adaptation to environmental heterogeneity. The forces responsible for their origin and maintenance are incompletely understood. Molecular characterization of inversion breakpoints provides insight into how they arose, and provides the basis for development of molecular karyotyping methods useful in future studies.

**Methods:**

Sequence comparison of regions near the cytological breakpoints of 2Rb allowed the molecular delineation of breakpoint boundaries. Comparisons were made between the standard 2R*+*^*b *^arrangement in the *An. gambiae *PEST reference genome and the inverted 2R*b *arrangements in the *An. gambiae *M and S genome assemblies. Sequence differences between alternative 2R*b *arrangements were exploited in the design of a PCR diagnostic assay, which was evaluated against the known chromosomal banding pattern of laboratory colonies and field-collected samples from Mali and Cameroon.

**Results:**

The breakpoints of the 7.55 Mb 2R*b *inversion are flanked by extensive runs of the same short (72 bp) tandemly organized sequence, which was likely responsible for chromosomal breakage and rearrangement. Application of the molecular diagnostic assay suggested that 2R*b *has a single common origin in *An. gambiae *and its sibling species, *Anopheles arabiensis*, and also that the standard arrangement (2R*+*^*b*^) may have arisen twice through breakpoint reuse. The molecular diagnostic was reliable when applied to laboratory colonies, but its accuracy was lower in natural populations.

**Conclusions:**

The complex repetitive sequence flanking the 2R*b *breakpoint region may be prone to structural and sequence-level instability. The 2R*b *molecular diagnostic has immediate application in studies based on laboratory colonies, but its usefulness in natural populations awaits development of complementary molecular tools.

## Background

*Anopheles gambiae sensu stricto*, the most important vector of human malaria in Africa, is the nominal member of a group of at least seven morphologically indistinguishable and closely related mosquito species [1,2]. Polytene chromosome analysis of this group, the *An. gambiae *complex, revealed an abundance of paracentric inversions [1,3], characterized by the breakage and 180 degree rearrangement of chromosome segments excluding the centromere. More than 130 paracentric inversions have been detected across the group as a whole, with 10 inversions distinguishing six of these species. Given the absence of morphological differences, these fixed chromosomal landmarks provided the first routine basis for species identification [1]. However, fixed inversion differences between species and populations are the exception in the *An. gambiae *complex. The majority of inversions remain polymorphic in natural populations. Although most species in the complex carry at least one polymorphic inversion, only two species--*An. gambiae s. s*. (hereafter, *An. gambiae*) and *Anopheles arabiensis*--possess most of the known inversion polymorphisms, potentially helping to explain their vast geographic and ecological distributions across much of tropical Africa [1,3].

**Figure 1:**
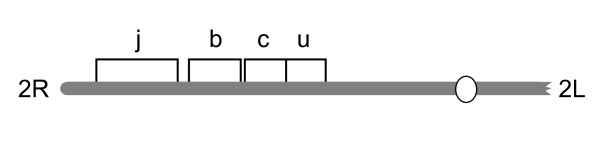
**Schematic diagram (after **[13]) **of common inversions 2Rj, 2Rb, 2RC and 2Ru on chromosome 2R in *An. gambiae***.

Cytological studies have also demonstrated the highly non-random occurrence of inversions along the five arms of the polytene complement in *An. gambiae *[1,3,4]. Contrary to the null expectation that they should be distributed among chromosome arms in proportion to arm length, inversions are highly concentrated on the right arm of chromosome 2 (2R) (Figure 1). Although 2R represents less than 30% of the complement, this arm is the source of 6/7 (86%) common, and 67/82 (82%) rare, polymorphic chromosomal inversions in *An. gambiae *and 18/31 (58%) common polymorphic inversions in the species complex as a whole--a highly significant bias [3,4]. Moreover, the distribution of inversion breakpoints along the 2R arm is not uniform. Inversion breakpoints not only cluster in particular regions, but also appear coincident at the cytological level at a much higher rate than expected by chance [4], suggesting that a nonrandom process could be responsible for their origin and/or maintenance. Intriguingly, Coluzzi *et al *[3] identified the central part of chromosome arm 2R, the area corresponding to polymorphic inversions 2R*b*, *bc *and *u *in *An. gambiae*, as being involved in independent interspecific inversions in the sibling species *Anopheles merus*, *Anopheles melas *and *An. arabiensis*, leading him to speculate that these parallel chromosomal changes may underlie adaptations to ecologically distinct larval breeding sites characteristic of these different species.

The abundance of inversions in the *An. gambiae *complex, and their nonrandom taxonomic, genomic and ecological distributions suggested that they may be playing more than a passive role in the diversification of these mosquitoes. Indeed, chromosomal inversions have been viewed as a mechanism for ecotypic differentiation in *An. gambiae *[3,5,6]. A recent model that could explain the spread and distribution of inversions proposes that through suppressed recombination in inversion heterozygotes, allelic combinations beneficial in particular environments are protected from recombination with other genetic backgrounds adapted to alternative ecological settings [7,8]. The maintenance of polymorphic inversions would thereby confer greater ecological flexibility to the species. Evidence consistent with an adaptive role for inversion polymorphisms in *An. gambiae *are the stable geographic clines in inversion frequency associated with climatic factors, such as aridity, replicated across Africa [1,9-11]; the microhabitat differences in inversion frequencies of indoor- or outdoor-resting populations, also associated with aridity [12]; the temporal cycling of inversion frequencies in concert with rainy and dry seasons [1,13]; and the heterotic maintenance of inversions in laboratory colonies [14]. Of particular relevance to malaria transmission and control is the connection between inversions and indoor/outdoor resting, as this mosquito behavioral response to aridity impacts the probability of vector-human contact and vector contact with insecticide-treated walls or bed nets [15]. Moreover, the process of ecotypic differentiation, potentially leading to speciation [5], implies restricted gene flow between populations and may be accompanied by other physiological and behavioral differences that could alter the efficiency of vector control efforts in unanticipated ways.

To date, only three inversions have been molecularly characterized in *An. gambiae s.l*., one on 2L (2L*a *[16]) and two on 2R (2R*j *[17]; 2R*d' *[18]). Based on detailed molecular analysis of the breakpoint junctions, no clear consensus has emerged on the likely mechanism of breakage, nor have precise mechanisms responsible for inversion maintenance yet been identified. However, characterization of these breakpoint regions led to the development of molecular diagnostics for 2L*a *[19] and 2R*j *[20], which allow much higher throughput karyotyping of natural populations than allowed by laborious cytological methods, and have facilitated ongoing functional analysis [21-23]. Toward the long-term goal of understanding mechanisms responsible for the origin and maintenance of inversions in *An. gambiae*, the breakpoint structure of inversion 2R*b *is presented along with a rapid molecular karyotyping method.

## Methods

### Assembly of 2Rb breakpoint proximal sequences

Using PCR primers designed from the chromosomally standard *An. gambiae *PEST reference genome [24]; [25], a fosmid library prepared from the *An. gambiae *BKO strain (2R*j+*^*b*^*cu*/*j+*^*b*^*cu*; [17]) was screened for clones that possessed unique (non-repetitive) sequence corresponding to the centromere-proximal breakpoint region of the 2R*+*^*b *^arrangement. (This strategy was part of a larger, ongoing effort to molecularly characterize other inversion breakpoints on chromosome 2R). Based on localization with *in situ *hybridization and sequence comparison, Fosmid clone 332D was identified as going to the breakpoints, and end-sequenced using the Big Dye Terminator v3.1 Cycle Sequencing kit (Applied Biosystems) and the ABI PRISM 3700 DNA Analyzer (Applied Biosystems). Sequences verified using Lasergene Seqman software (DNASTAR) were submitted to GenBank (accession numbers HN153245-HN153246).

End-sequence of fosmid 332D was used to walk toward the 2R*b *proximal breakpoint using trace sequence reads generated from whole genome sequencing of the *An. gambiae *S form (Pimperena strain; Lawniczak *et al*, submitted). The Pimperena strain carries the opposite (inverted) orientation of the 2R*b *arrangement (*i.e*., 2R*b*/*b*). Pimperena trace sequences matching sequence from fosmid 332D were used to initiate an iterative BLAST procedure in which the Pimperena trace archive was queried and contigs from this 2R*b *background were built using mate pair information and sequence similarity. These 2R*b *contigs were compared to the PEST (2R*+*^*b*^) assembly to find evidence of the rearrangement breakpoint. Highly repetitive regions that could not be assembled manually were excluded. Manually assembled 2R*b *sequences flanking the putative breakpoint were used to identify scaffolds assembled independently during the *An. gambiae *M (Mali-NIH, 2R*bc*/*bc*) and S genome sequencing project [26,25] and compared to the PEST genome.

### Mosquito sampling and cytological determination of karyotype

Collections of indoor resting *An. gambiae *were made by spray catch from Mali and Cameroon. Samples from Mali were collected from seven villages in the southern part of the country in Aug-Sep 2004, as previously described [20]. Samples from Cameroon were collected from five villages in a forest/savanna mosaic zone in the eastern part of the country in Sep-Oct 2009: Gado-Badzere and Zembe Borango (4°58.435'N, 13°2.403'E), Nkoumadjap (4°21.575'N, 13°38.391'E), Mayos I (4°20.505'N, 13°33.490'E), and Daiguene (4°46.608'N, 13°50.668'E). Mosquitoes were sorted morphologically to *An. gambiae s.l*. and by gonotrophic stage. Ovaries of semi-gravid females were dissected and placed into a micro-tube containing Carnoy's solution (1 part glacial acetic acid, 3 parts ethanol); the carcass was placed in a correspondingly numbered micro-tube and stored over desiccant for DNA-based analysis. Preparation and scoring of polytene chromosomes followed [20].

### PCR determination of karyotype

Genomic DNA was isolated from individual mosquitoes using the DNeasy Extraction Kit (Qiagen, Valencia, CA). *Anopheles gambiae sensu stricto *and its molecular forms were identified using an rDNA-based PCR diagnostic assay [27].

Sequence comparisons between the PEST (2R*+*^*b*^), Pimperena (2R*b*), and Mali-NIH (2R*bc*) genome assemblies were used to design primers to differentially amplify alternative arrangements of the *b *inversion. Diagnostic primers include: b-For (5'-CGGGAGCAAAGATAAGTAGCA), b^+^-Rev (5'-CCGGATAATCGACGCTCTAC), and b-Rev (5' AACCCTACCATATACCAGTACCAACG 3'). The 25 μl PCR reaction contained 20 mM Tris-Cl (pH 8.3), 50 mM KCl, 1.5 mM MgCl_2_, 200 μM of each dNTP; 5 pmol b-For primer, 2.5 pmol b-Rev primer, 5 pmol b+-Rev primer, 1 U Taq polymerase, 5% DMSO and 1% BSA. PCR was performed on 1 μl genomic DNA template using an initial incubation at 94°C for 2 min, followed by 35 cycles of 94°C for 30s, 58°C for 30s, and 72°C for 45s, concluding with a 10 min incubation at 72°C and a 4°C hold. Mosquitoes from established colonies with known karyotypes were used as PCR controls. These included Mali-NIH M form (2R*bc*; 2L*a*) and Pimperena S form (2R*b*; 2L*a*/*+*^*a*^) available from MR4[28], and colonies selected at the University of Notre Dame to be homokaryotypic for chromosomal inversions on 2R: CAM M form (2R+; 2L+, derived from parental colony Yaounde from Cameroon), KIST S form (2R+; 2L+, derived from parental colony KISUMU from Kenya), and SUCAM M form (2R+; 2L*a*/*+*^*a*^, derived from a cross between CAM and Sua2La [22]).

## Results and Discussion

### Sequence assembly across the 2Rb breakpoints

**Figure 2:**
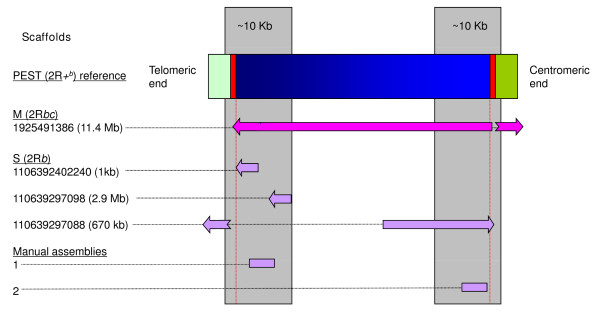
**Schematic diagram of scaffolds analysed in M and S relative to the *An. gambiae *PEST reference**. Horizontal rectangle (labeled at left) represents genomic sequence from the PEST reference genome, with green shaded sections indicating flanking sequence at the telomeric and centromeric ends, red sections indicating repetitive sequence at both breakpoints, and the blue section indicating the 2R*+*^*b *^arrangement. Horizontal arrows indicate the relative position (not to scale) and orientation of assembly or manual scaffolds used in the analysis. The scaffold number (if applicable), approximate length, and source (*An. gambiae *M or S genome sequence) is indicated at left. Vertical gray rectangles highlight the ~10 kb breakpoint regions whose sequence was compared between the PEST, M and S genomes.

Three genome assemblies were compared to infer the molecular organization of the 2R*b *rearrangement breakpoints. The *An. gambiae *PEST reference is homokaryotypic standard (uninverted) for all chromosomal arrangements including 2R+^*b *^[24,29], while the S and M form assemblies were derived from colonies (Pimperena and Mali-NIH, respectively) carrying the opposite (2R*b *inverted) arrangement [26]. Sequence spanning the breakpoint regions of the 2R*b *inversion were assembled manually, using Sanger trace reads and mate pair information available from S form genome sequencing on Vectorbase [25]. The query sequence used to seed iterative searches of S traces was end-sequence determined from a fosmid clone (332D) shown to hybridize *in situ *to the centromeric end of the 2R+^*b *^arrangement. The two resulting manual trace assemblies (available as additional file 1), representing the last ~10 kb of sequence at both ends of the rearrangement, were validated by comparison with scaffolds generated from independent automated whole genome shotgun (WGS) assemblies of M, S PEST (Figure 2).

**Figure 3:**
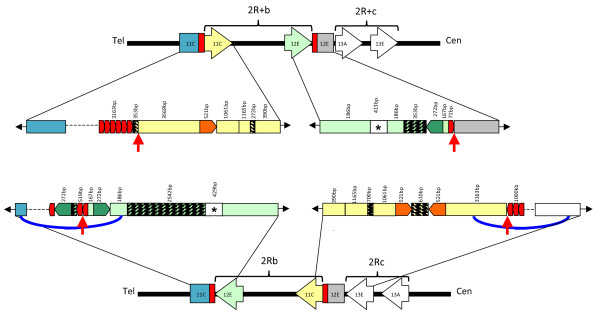
**Structure of the 2R*b*/*+*^*b *^breakpoint junctions**. At top and bottom is a schematic overview of the 2R*b*/*+*^*b *^and 2R*c*/*+*^*c *^arrangements as represented by the PEST and M reference sequences, respectively. Horizontal black bar represents a segment of chromosome 2R. The relative position and orientation of chromosomal arrangements is indicated by labeled brackets, and centromeric/telomeric ends of 2R are indicated as Cen and Tel, respectively. Shaded arrows indicate the orientation of the arrangements, and labels inside the arrows (*e.g*., 11C) provide the cytogenetic subdivision in which the breakpoint junction occurs. Blue and gray boxes labeled with the corresponding cytogenetic subdivision represent flanking sequence outside of chromosomal rearrangements; red boxes represent repetitive DNA. The central part of the diagram provides a more detailed structural analysis of the color-coded breakpoint regions. Throughout, color is used to indicate homologous sequences between alternative arrangements, except for rectangles filled by patterns, which represent exclusive insertion events. Horizontal blunt arrows shaded in olive green and orange are sequences present once in the 2R*+*^*b *^arrangement that have been duplicated into a palindrome in the alternative 2R*b *arrangement. The red vertical arrows represent the putative breakpoints, positions where unique sequence ends and repetitive sequence framing both ends of the arrangement (red blunt arrows) begins. Black arrows at the ends of each diagram represent continuing chromosomal sequence; dotted lines represent gaps in the assembly. Blue curved lines represent sequence linked by mate-pair information. Asterisk framed by a white box indicates the chromosomal region targeted by the PCR diagnostic assay; see text and Figure 4 for details. Not to-scale.

Mate-pair information verified the linkage of sequence within the inversion to flanking sequence outside both breakpoints in the manual assemblies. However, neither breakpoint could be manually assembled without gaps, due to the presence of highly repetitive sequences (Figure 3). Gap-lengths were estimated based on mate pair and clone insert-size information. Both breakpoint regions also contain gaps in all three WGS assemblies (M, S and PEST). Scaffolds 1106392397088 (S assembly) and 1925491386 (M assembly) span the proximal (centromeric) 2R+^*b *^breakpoint across gaps and into unique flanking sequence. Neither M nor S WGS assemblies produced scaffolds that spanned the distal (telomeric) breakpoint.

### Molecular organization of the 2Rb inversion breakpoints

Assemblies of the 2Rb inversion breakpoints from Mali-NIH (2Rbc) and Pimperena (2Rb) traces were nearly identical, with only minor SNP and insertion-deletion differences. A schematic diagram of their molecular structure, in comparison to the corresponding regions of the uninverted chromosome, is provided in Figure 3. Together, these data reveal that the 2Rb inversion encompasses 7.55 Mb and extends from subdivision 11C to 12E on the cytogenetic map of PEST (position 19,023,925 to 26,758,676 on 2R; red arrows in Figure 3).

Outside of and immediately flanking these breakpoints on both 2R+^*b *^and 2R*b *arrangements is a repetitive structure comprising tandemly arrayed copies of unit length ~30 bp, (ACTTTTGCGATTGTCGCAAAAACTTCTGCGA)_N_. At the telomeric end of the 2R+^*b *^arrangement of PEST, this tandem repeat structure extends for at least 3.2kb (i.e., >100 tandem repetitions) and is flanked by a ~10 kb assembly gap. At the centromeric end of 2R+^*b*^, the repetitive structure is 72 bp in length. The alternative 2R*b *arrangement in both Mali-NIH and Pimperena also was flanked by the same tandem repeat sequence. At the telomeric breakpoint of 2R*b*, the tandem repeat sequence spans 519 bp and is embedded in a palindrome, flanked by a ~2.5 kb assembly gap. At its centromeric end, the 2R*b *breakpoint also abuts at least 1 kb of the same tandem repeat, but the highly repetitive nature of this sequence caused gaps in both WGS and manual assemblies.

The position of these tandem repeats, presumably at the precise breakpoints of both arrangements (2R*b *and 2R+^*b*^), implicates the tandem repeat in the generation of this chromosomal inversion, although the ancestral-descendant relationship between the alternative gene orders based on these data is ambiguous. Other molecularly characterized inversion breakpoints on 2R in *An. gambiae *also possess flanking repetitive sequences, but only on the derived arrangement: 2R*j *is flanked by nearly identical 14.6 kb complex inverted repeat structures reminiscent of segmental duplications, while 2L+^*a *^is bordered by homologous repetitive elements [17,19]. In neither case were these repetitive sequences found adjacent to either breakpoint of the ancestral arrangement, in contrast to the situation for 2R*b*. Either the 2R*b *or 2R+^*b *^arrangements could have arisen via non-allelic homologous recombination between flanking tandem repeats on the ancestral chromosome, leading to inversion of the intervening sequence. Notably, two unrelated palindromes with short internal spacers were present at the breakpoints of the 2Rb inversion in both Pimperena and Mali-NIH 2R*b *arrangements, one at each end. As the arms of both palindromes involve sequences apparently present only once in the PEST genome (near each breakpoint), it is tempting to suggest that the 2R+^*b *^arrangement in PEST may be ancestral.

### Gene annotations adjacent to the 2Rb breakpoints

Gene predictions were compared between the 2R*b *and 2R+^*b *^arrangements, within the ~10 kb region immediately internal to the breakpoints. Most of the sequence consisted of transposons and low complexity sequence. There were no genes annotated in the 10 kb region proximal to the centromeric breakpoint of 2R+^*b *^in PEST. At the telomeric end of this arrangement, one gene has been predicted of unknown function (AGAP002299, annotated as a conserved hypothetical protein). This gene has putative orthologs in other mosquitoes (*Culex quinquefasciatus*, CPIJ008031; *Aedes aegypti*, AAEL007792) and *Drosophila melanogaster *(CG18635). The corresponding gene at the centromeric end of 2R*b *contained several SNP differences and a deletion in the predicted 5' UTR. Although limited EST evidence suggests that this gene may have alternative transcripts, potential functional consequences of sequence differences in AGAP002299 between alternative arrangements (if any) remain to be investigated.

### Molecular karyotyping by PCR

**Figure 4:**
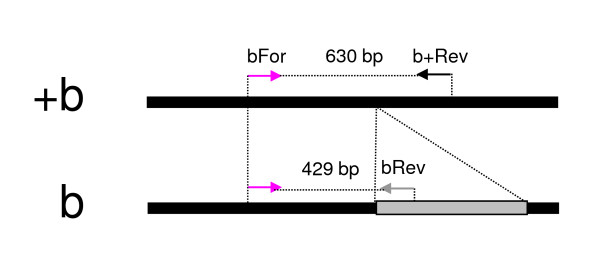
**Schematic diagram of the three-primer PCR assay for molecular karyotyping of 2R*b***. The white box with an asterisk in Figure 3 is represented here. Areas common to both arrangements are connected by dotted lines. The grey box represents an insertion exclusive to the 2R*b *arrangement, to which primer bRev anneals. Primer bFor is a universal primer that anneals to a region common to both arrangements. Although +^b^Rev can anneal to both arrangements at different distances from bFor, size limitations on successful PCR amplification restrict the product to a 2R+^b^-specific fragment of 630 bp. In combination with bFor,primer bRev amplifies a 429 bp sequence diagnostic of the 2Rb arrangement.

Extensive lengths of repetitive DNA and associated assembly gaps precluded a molecular karyotyping strategy that depends upon PCR amplification across inversion breakpoints. Instead, a PCR assay was developed that exploits an insertion-deletion difference between arrangements, as close as possible to the breakpoint (~1 kb; Figure 3). The assay employs three primers, one of which (bFor) is a universal primer that anneals to both arrangements (Figure 4). The second primer, bRev, was designed to anneal to a 2.5 kb sequence exclusive to the 2Rb arrangement. Together, bFor and bRev should amplify a 429 bp fragment when 2Rb is present. Although the third primer, +bRev, can anneal to both arrangements, successful PCR amplification with this primer is expected only from the 2R+b arrangement, on which the distance spanned by +bRev and bFor is 630 bp (the corresponding distance between these primers on the 2Rb arrangement exceeds 3 kb).

As a first step in the validation of this assay, its performance was tested on at least 25 mosquitoes (50 chromosomes) sampled from each of five different *An. gambiae *laboratory colonies of known and monomorphic 2R*b *karyotype (determined from polytene chromosome banding pattern), originating from geographic locations as diverse as Mali, Cameroon, Liberia, and Kenya: Mali-NIH M form (2R*bc*/*bc*), CAM M form (2R*+*^*b*^/*+*^*b*^), SUCAM M form (2R*+*^*b*^/*+*^*b*^), KIST S form (2R*+*^*b*^/*+*^*b*^), and Pimperena S form (2R*b*/*b*). Without exception, PCR amplicons of the expected size were generated. Moreover, when DNA was mixed in 1:1 proportion from mosquitoes carrying 2R*b *or 2R*+*^*b *^karyotypes prior to PCR, both bands were amplified (suggesting that the assay is capable of detecting 2R*b*/*+*^*b *^heterozygotes). Additionally, at least 15 mosquitoes from an *An. arabiensis *colony (Dongola) selected to be homokaryotypic for 2R*b *(*i.e*., 2R*b*/*b*) were tested, and each generated the expected 429 bp PCR fragment (and only this fragment), consistent with other evidence that the 2R*b *inversion in *An. arabiensis *and *An. gambiae *shares a common origin (*e.g*., see [30]).

As a second step of validation, the PCR assay was performed using *An. gambiae *sampled from natural populations in southern Mali and eastern Cameroon. Females at the appropriate gonotrophic stage were karyotyped based on polytene chromosome banding pattern, and these cytogenetic results were compared to those obtained from molecular karyotyping of the same specimen. Of the 267 mosquitoes whose karyotype could be determined both cytologically and molecularly in the overall sample, 223 (84%) yielded congruent results (Table 1). In the Cameroon collections were five *An. arabiensis*, of which two were successfully karyotyped as 2R*b*/*b*. Their molecular karyotype was congruent, as judged by the expected 429 bp amplicon observed for both mosquitoes.

**Table 1:** Performance of the 2R*b *molecular karyotyping PCR assay in field collections of *An. gambiae*

	Molecular karyotypes congruent with banding pattern	
	
Banding pattern	Cameroon	Mali
**2R+**^**b**^**/+**^**b**^	67/70 (96%)	14/31 (45%)

**2R+**^**b**^**/b**	55/65 (85%)	16/22 (73%)

**2Rb/b**	30/37 (81%)	42/42 (100%)

In the Cameroon sample, departures from the expected molecular results revealed no obvious trend apart from the fact that the PCR assay appeared to be less successful at accurately diagnosing the 2R*b *arrangement, especially in 2R*b*/*b *homokaryotypes. The frequency of other 2R inversions observed in this sample (2R*c*, 2R*d*, and 2R*u*) was very low, ~3%. By contrast, cytologically defined 2R*b*/*b *mosquitoes from Mali were invariably recognized as such by the PCR assay. However, the accuracy of the molecular method apparently declined precipitously for 2R*b*/*+*^*b *^heterokaryotypes and even more so for standard (2R*+*^*b*^/*+*^*b*^) homokaryotypes from Mali. A more in-depth analysis of the full, cytologically determined 2R karyotypes of mosquitoes responsible for the discrepant molecular results revealed a remarkable insight. This insight is founded on the recognition by Coluzzi and coworkers that inversion 2R*c *is found almost exclusively in combination with 2R*b *and 2R*u*, as 2R*bc *or 2R*cu *in Mali, where chromosomal polymorphism on 2R is very high [3,13]. Taking this observation into account, mosquitoes from Mali yielding incongruent molecular results--all 17 (100%) of the 2R*+*^*b*^/*+*^*b *^homozygotes and all 6 (100%) of the 2R*b*/*+*^*b *^heterozygotes-- also carried the 2R*cu *arrangement (Table 2). By convention, mosquitoes scored as 2R*cu*/*cu *based on chromosomal banding pattern carry the banding pattern typical of the standard arrangement with respect to other 2R inversions (*i.e*., 2R*+*^*j*^*+*^*b*^*cu*/*+*^*j*^*+*^*b*^*cu*; [13]), and thus should be diagnosed molecularly as 2R*+*^*b *^(*i.e*., presumably two copies of the 630 bp PCR amplicon, given the expected 2R*+*^*b*^/*+*^*b *^karyotype). Similarly, mosquitoes scored as 2R*b*/*cu *(*i.e*., 2R*+*^*j*^*b+*^*c*^*+*^*u*^/*+*^*j*^*+*^*b*^*cu*) should be diagnosed molecularly as 2R*b*/*+*^*b *^heterozygotes. Instead, the presence of the 2R*cu *arrangement was perfectly correlated with presence of a 429 bp amplicon that is normally diagnostic of the 2R*b *arrangement. Sequencing of this unexpected amplicon verified that it matched the 2R*b *sequence between the bFor and bRev primers (100% sequence identity), thus ruling out the possibilities that the fragment was an unrelated sequence fortuitously close to 429 bp in length, or a foreshortened segment of the 2R*+*^*b *^chromosome. Taken together, these results are consistent with the hypothesis that the 2R*cu *arrangement may be derived from an ancestral 2R*b *chromosomal background. This implies a secondary rearrangement from 2R*b *back to 2R*+*^*b *^on chromosomes whose banding pattern appears to be 2R*+*^*b*^*cu *through the microscope. The arrangement of tandem repeats containing the same core sequence at opposite sides of the 2R*b *breakpoint could provide the substrate for successive rearrangements through breakpoint reuse. However, it should be noted that this hypothesis depends at least in part upon an interpretation of some karyotypes that are not cytologically distinguishable (*e.g*., 2R*bc*/*u *versus 2R*b*/*cu*; Table 2), and it requires validation by further molecular investigation.

**Table 2:** Molecular karyotyping discrepancies in relation to banding patterns on 2R in Mali

Source of anomalies based on 2R*b *karyotype	Complete 2R karyotype	Exp. molecular karyotype	Obs. molecular karyotype
**2R*+***^***b***^**/*+***^***b***^** (N = 17)**	**2R*j+***^***b***^***cu*/*j+***^***b***^***cu *(N = 13)**	**2R*+***^***b***^	**2R*b***

	2R*+*^*j*^*+*^*b*^*cu*/*+*^*j*^*+*^*b*^*cu *(N = 3)	2R*+*^*b*^	2R*b*

	**2R*+***^***j***^***+***^***b***^***cu*/*+***^***j***^***+***^***b***^***+***^***c***^***+***^***u***^** (N = 1)**	**2R*+***^***b***^	**2R*b***

2R*+*^*b*^/*b *(N = 6)	2R*j+*^*b*^*cu*/*jbcu *(N = 4)	2R*+*^*b*^/*b*	2R*b*

	**2R*+***^***j***^***b+***^***c***^***+***^***u***^**/*+***^***j***^***+***^***b***^***cu *(N = 2)**	**2R*+***^***b***^**/*b***	**2R*b***

Application of this PCR assay in Mali in conjunction with cytogenetic analysis, raised the intriguing possibility of 2R*b *homoplasy through breakpoint reuse. However, it appears that the PCR assay as presented here has limited application by itself, for molecular karyotyping of 2R*b *in natural populations of *An. gambiae*, even in Cameroon where the degree of 2R chromosomal polymorphism is low, due to a relatively high rate of 2R*b *"miscalls". The rate of miscalls is based on the assumption that the cytogenetic analysis was error-free, which is unlikely. Thus, the miscall rate may be over-estimated. Nevertheless, it is possible that high repeat content near the 2R*b *breakpoints is associated with relatively high genetic instability and sequence rearrangement, which can result in elimination or alteration of primer binding sites as well as unexpected changes in amplicon length due to insertions and deletions. On the other hand, the PCR assay yields results that are perfectly congruent with cytology in all laboratory colonies tested thus far, suggesting that the diagnostic assay will be useful in experimental manipulations and crosses where rapid karyotype analysis of living mosquitoes, both males and females across all developmental stages, is desired. Moreover, the 2R*b *PCR assay may prove useful in Mali in the future, in combination with yet-to-be-developed molecular diagnostic assays for other arrangements on 2R.

## Conclusions

Elucidation of the molecular breakpoint structure of the 7.5 Mb 2R*b *inversion points to the involvement of repetitive DNA-- specifically, extensive tandem arrays of short unit length flanking both breakpoints-- in the rearrangement process. Although the ancestral-descendant relationship between standard and inverted arrangements is uncertain, molecular karyotyping based on a newly developed PCR diagnostic assay suggests two things. First, the 2R*b *inversion shared between the sibling species *An. gambiae *and *An. arabiensis *has a common origin. Second, the polytene chromosome banding pattern indicative of the 2R*+*^*b *^standard arrangement may have arisen twice through breakpoint reuse. Sequence instability and high repeat content near the breakpoints complicate the application of the PCR diagnostic assay for molecular karyotyping of natural populations, although the assay represents a novel and powerful tool for functional genomic studies of 2R*b *in laboratory colonies, and may hold promise for future field application in combination with other molecular tools. The current impediments to analysis of inversion breakpoints posed by repetitive DNA may be overcome by powerful new technologies [31], such as single DNA molecule platforms capable of mapping and even sequencing repetitive DNA, enabling further insights into the origin and stability of 2R rearrangements in natural populations of *An. gambiae*.

## Competing interests

The authors declare that they have no competing interests.

## Authors' contributions

FHC and DMS conceived the study. NFL and FHC designed and coordinated the study, with the assistance of SJE, SFT, CC and NJB. NFL, DMS, AAR, KRR, DAB, and MVS performed molecular, cytogenetic and/or computational experiments. NFL, DMS, and FHC analysed the results. NFL wrote the manuscript, with contribution from NJB. All authors read and approved the final manuscript.

## Supplementary Material

Additional file 1 **that provides manual assemblies prepared from *An. gambiae *S proximal and distal 2Rb breakpoint regions as described in the Methods section, and the trace mate-pairs from the S genome, which cross both breakpoints**. Click here for file
