# Supplementary material for: Breakpoint structure of the Anopheles gambiae 2Rb chromosomal inversion
Source: Malar J. 2010 Oct 25;9:293. doi: 10.1186/1475-2875-9-293 (PMC2988034; doi:10.1186/1475-2875-9-293)
Supplement: Additional file 1 — that provides manual assemblies prepared from An. gambiae S proximal and distal 2Rb breakpoint regions as described in the Methods section, and the trace mate-pairs from the S genome, which cross both breakpoints. [file 1475-2875-9-293-S1.PDF]

This manual assembly from the S WGS lies inside the Distal breakpoint and matches sequence on the PEST chromosome (19023925-19027916)

>1(4441bp)
GGCAAATGGCCAAATTTCAGTAGCTTTGAGATCTGTTAGGAGACATTCACGAACTCTTGTCTGATGATTATAGTATCGT
TATTTCTCTCTGTGTTTCTAAGCATCGCACAAGCACATGCCCAACATAAAACCGCTTGTGTATTCTGCTCCCTGGAACAC
CTTACTGTGGACTGTGTCGTGAAAGACTATCGGGACCATGTTCACACTATCGCCGTAAAAAACAAAGTTATCTGAAACC
GCGTTGAGGCACGCCAGCAGCAAGTACTTGCAACAAACCGCTTATCGCACCGGCTGGGTGACAAACACGGTCACCCGT
TGAGTCAGAAGGGGCGAAAGGCTTTCCGATAACGCTACACGCACCGAAAGCGATAGCACGCACACACACACACACCCT
CGTTGGCGCGCTAGACCTAACATCAATCGTTTGGCGAGCCAGTTACCGCAAAAGAGAGTTTGCACGATGGACGCCCTC
GATGGTGTGCCACGATCCGTGCTCGGCTACCCGGTCAAGACGCGCACCCAAATAGTGCACTATTTGGGCCATGGCCACC
TTCCTGGTGCCGGTGATCTTCGAGCTGATGTTCGATGTTCGATGTCAAACACACCCATTCGTACTCTCTCGCTCTCTCCC
TTCAGCTTCAGCCGACGCATTTTCTGGAGCGTCGAAGCCCTGTTCCACGACAAAGGCTCCTACGGCCGTGCGCAAGCCG
TCGCCAAAATAACGGAAACGTCTCCCTGCGAGCTGTACCACTTTCTGCAGGCGTTCCTGCAGGCCGCCCCCCAGATGAT
GCTGCAGCTGTACATCCTGCTGCGGGACGGCACGTTCCGCAACTACGACACGGTCACGTGCAGTGATCAGCGTCGTCTG
CTCCTTCCTCACGATGACCACCATCATTACCGGCTATCAGCGCTTCGAAAGCCAGAGATCGTTGGCCGCTGCTATCCGT
GAGCACACCCGAGCAGCACGCACGTCGCAGCAGCTGCTACGATCGACGAGCATGCGTGTCGCGCTGCCACACCGTCTA
TAGCGCCGGAAGCGGATCCCATCGTGCCGAACATTATGCGCAACTTTTACTCGCGCTACGGCAGCGAAGGGGAGCGTG
AATCGACGGCAAATGAGCGAGTGGCGGTGCCGACCGCCGTGCAAAGTCCCCGGAAAACGCTCAACGTCGGTGCGTTCA
ACTTCGATCGGCAGCAGTCGAAAGCGCACCAGGATCCGTACGTCAACTTTACGGACGATGATAAGCTGCAGGAGTTTA
GCGGGCCGCCGGACACGGTGGATCGCGCTGGCTCAAAGGCTCGACCATCGGGGCGTAGCGTTAGCTTCAAGCTGTGGG
ACACGGTCGAGGAGCTGCAAGCGATGGACCATTATCTGCGCAGCACGGAAGACGTACGCGGGTACGCTACCGACGAC
AGTGACGACGAGTACCTCAAGCCGGACGGGTTCGATGCGGTACAGAAGCGGCCAGCCCCACCAACACCGGAAGCGGC
GGCCGGTATGTTTCACCGCGTGTCCGTCTTCCGCCACATGCTGCTCTTCAATGCGGAAGCGTTCATAAAGGAGAAGGTG
CCCCGGCTGCCCGAGGGCATGTTTGACCATGCGAATCCCAAGGAAGGAAGCCGGAAGGCCCAAGGGACGGACGATCA
GACGGATGGAGATGCGATATCGCTACCCTCCCGGCGCCAAACGATCGTGGGGCTCGAGCAGGACGATATGGTAGGCAA
GACGGTACTGTTTCTCGGCTGGGTAATGTTTCTGCTGATGCGCATGCTGGCCCTGTCCACGTTCTACGTGTTTTTCCCAC
TGTACTTCTGGATGCTGGTGGCCAACCACTATCTGCTGATGATCGCCTGCATCGTGTACGAGGTGCGGCTGCACGAAAA
GCTCGAGCGCTACTTCTTCTACCTGTTTCTCGCCTACATGTACGTGTTTTCGCTGCTCGAGTTTAAGATACGGTTCGTGC
ACGTGCGCTGGTGGTACGTCGGGTACATTGCGCTGGTGCTGGCCGAAAATGTGGCCTCGCTGGTGCTGTGGTACAACTT
GGGTGCGTTCGAGTCCTGGTGGTTTTACTTCCTGCACCACACGATCGTGGCGAGTGGGTCGTTGAGCGTGCTGTGCTTCC
TGTTCTATTACGCGTTTCTGCGCCCCAAGGATAAGGTGTTGTTCGTCAATGAGGACTAGTGTTGGGGTTTGCTAAGTGA
GCGTCCCAACGTGCAAAGGTTGCTCTCTAGGGTAAGCCAGTTTGTGTGTGTGTGCGTGTGATTTTAGTTCATTAGAGAT
ATTTATTTAAACTGATGTATTAACTTTGCGATCTATTACAACTATTTTAGTGCTTTAATTTTTGTAACCCATTTTGTTTAC
TAACGTAAGACGCCCAGCAATAAACAATTGTGTAGCAACGGTAGTAGAAAGGTGATCGCCACCGATAAGTGAGAGTGA
TAATAGCTTTCAGCGGTATCCTAAATGAAGCATTACCGGTGTAATTAAATTTTACTACAACCAAACGACCGATCTTCCC
CGCCTCCGAAACATCCTGCCCTAGACCATGCCACGCTGCCAATCGAAAGATTAGCTAAAAATCATTACACCTTGCAGCA
AAGTAAGACATAACCGTTTGCTGTGCCCACAACTTGGCAGCCTCCGACCCTTTTGGGAAGCTTTGCCATTTACAAGAGC
AAAAATTGGCACTCGGGTGACAACCATTTCGCTCGCCGCTTGCTGTCTCGTTCCCGTTCTTTTCCTATTTGGAGCCCTCA
AAAAGCCCCGCCACCGAGCTCGAGCGCAAAATCTCAAAAAGTCATAATCCTCCGTGCCATCGTGACTGGAATTTCATG
CGTTTTGTTTCCGGGGTGTTTTGCCTGCCCCCGGTCTCCCACCCTGTGGGAGGGTATAACCTTGCAATGCCCCACTAAAT
ATTAACAACTTTCCAGATAATGCGCCACTGCGAAACGAAACAGAGTGCGTGCGGCCCAACCCAAAAACCGATCCGATC
CGATTGACGGAAAAAACGACAGAGACTATTAATGTGACAGGAGGGATTTGTCAGGAGGGAGACAAATGCTGATAGAG
CGAGAGGATTGCGGGATGGTCGTAATGTTTTAATCTATTAAATTGATTAGAGATAAATATTTTATGAACGCTTGAATCA
ACTTCAACCAATCATATCTTGCAAACGGGCCGGGGCTTCCAGCCCGTGTGTCGTGCCGTTCCAGCACGCGATTCACAAA
AGCATCGCCATCAACCCCCCCCACCCCCAACGTGGTGCTAATGGCGGCATTCACACATTCTCCATAATGTTGGGCCTTT
CAAGGGGAAGTGATTTTCCGGAAAGAGTTTTAGCACCACCACCAACAGCAGCACCAGCAGCAGCAACGACAAGAACA
AGGAAAATAAAACGTCAACATGTTCCGGGAATGAATGGCGTACCACTGCCACTGGTGGCTGATGGTAGTGTGAAGCAT
TGCACGCGGTAGGCAACTTTATGAATGGAGCTCACTTTTATTACACATCCACTGTGGGTGGGTGAGACGAAATGGGTAA
TCGGAAAATGCAACGAAGGCAAGAACAAGGATGTTTGAAGCCCGTTGAATATCCGGAAGCGTATCATTGTTCGCTAAT
GGTGCACGGCAAGTTGCAGGAAAAGCTGTTCAGCACACATTGCTGCTAAATTGCGTTGCCTATTACTGATCTTGTGTTT
CGGACTGGTGCACAATTGACGATATTTAAGACACACGCGGCTCTTCTGTATAAACCACTTATATTCTAATATAAATAAC
ATATATTCTACAGGACTGTTGTGTGTGTCGGGTCGCGGCCTATGATCGAGTGCCGACCCTCGGTAGCCCGATCGCTCCC
AAAGCCCTTAACGGCCATTCGGACGTCGGATGCTGTCAGTTGCACGACAGCATCGAGTCGCGGTTACACTGCACCGTTT
CTGTACACAACATCTCCCTCTCTTAATTCAGCTGGTACAGCTGCAACCTGCGCGACGCCCTTCTATCACGAGAAGAGCG
GCGAGGTGGACCATTCATTTGTTCACGTGCGCAGACTATGCTAGGGGAATGTTGTGCCACGATGGCTAAAACTGGGTTT
GCTAAAATGCTAAAAATTGTGACGGCTATTTTGTTCCAGAAACTCTACCCTCAGCCATTAAAACATCCTACCGTAAATT
AATACCATTATTTTCCATTCTTGTATCACTTAATTGCTAGCACTTCTTTGAGACATGTACCCTCTCGCCAGGGATCCTTTA
ACTCATATGCCCGTTTATAAATCATTATTTACGCCTACGCTAAGGTTCTTGCGACCTCACGTGTCTCCCTTTTCTTTGGTA
CGCTTCGTGACAAATTATCGGCGAGGGCTGTTTACGAAGGTAATTGGCGCTATCTATTCGACCATAATTACCATAGCTA
TATAATCTGCGCAGACTCCCATTTTTGC

This manual assembly from the S WGS lies inside the Proximal breakpoint and matches sequence on the PEST chromosome (26747166-26758676)

>2(4576bp)
CGGGAGCAAAGATAAGTAGCAGTAGTAGTGATAGCAGTAGCAGTAGTGATAGTAATATTGTACTAAAAATAGACATCT
ACATGTGAGGACGTCGATGTGACACGTGATGGACATTGTACAGGGTGGGCTTGTGCACACCGGGTGCTGCTAAATGGA
AGCTGGAAAACCAAGACATTAGACTATATACAATTAAGCCAGTGGAATTGTTGTTTATCTTTCTCTCGTCTCCTCTCTGC
TCCACTACACGCTACGGGAGGGGAGGGGGTGTAATCATGGTTAAGGACCAGGGGCAGGGAAAGAGTATATTTATTGAT
AACCCGTCACGTTCGGAAATGTTATTCTTCCTTCGGATTTGTCACAAGTTTCTATGAAAAATAAAACTTTGAACTAAAGT
GTGGTTACGCGTTGGTACTGGTATATGGTAGGGTTGTAGTTGTATCTTGAATTGGCGATTGGTACATATATATGTAGTGT
ATTTGGTTTTACCTGTGACCTTGACGATGCGAGAGCCGATCAATTCTGGTATTGAAGCAATATTTGTGCAATGCTGGTG
GTTGCCGTTCCGTGCCGTTTCTGATGTGGCCATTTGATCGCCATGAGGATCAACGGTTATGAAGCCAATTTCAAACGAG
CGAAAGTGAAAGAATAATTCCGTACCGTAAATTTGATTTTTTTTATCAAGTGTGATACTCCAATGTTGATCTCAACCCGA
TGTAAGGTGGTTTTAACGGCCATATGAGATAATGTACTCATGTGTTAAAAAATGTCGCATTTTTTTCTTTCCTGATATTC
CCGAAAATTGACCCATAATTCAAATACGATTTGACTATTTTCATTGATTCCTCATACAACGCATGGAAAGAGGTAAACT
GGCTTCGCCAATTCATTCATCTCCGCTCCCAACCATTCATCACCCGAACCACCCACAATCTCATCATTCCATCCACATGC
AACGACATTTCCCTCTTCTTTAACAGCAAAGGTCTCAACCGAAGCTTCTACAGACTGCTGCTTTCCATTTTCCTGTTTCTT
CTTCTTCTATTTGCCGTATCCATCTACCTGGTCATACCAGCCAATGCAAGCTTTAGCGTCTTTTTTTAGTACCACACACCC
GGCTGGCCAGTCCTTACTACGAGGGGACCGTCTATATGAGGCTTAAACCTATGACGGGCATGATATTATCATCCTGTAT
CCAGTGGCGGATCTAACGGTAGGCGGACTAGGCGGTTGCCTGGGGCCCCGCCGATTAAGGGGCCCCGTAGATCGCCAT
TCTATAGTATGCCGTATGGACAGAGTACCAGCAACAAGGGCCCCCGGGACGTTGGGGCCCCGAGGCTGGCGAATCAAT
TTTAGAGCCTGTGACTGATGATCGTAGGGCCCCCCGACGGCGTTCAAACTCTCAACAGCCAGTTTAATGCCGCTAACAC
CCTCCCCCCCCCCTCCTCCCGGACCACCATTTACCATTTACAGGGGGCCTCAATTCGTCTTACCGCCAAGGGCCCCCGAT
ACCCTTAATCCGCCACTGTATGTATCCTGACCACTCTGACCACTGGGTGGTAGGTTTTTTCCTTTTTTGGGGCCCCTCAA
GACCACGGGGCCCGACGCGGCCGCTTAATTTGCTTAGCGCTTATTCCGCCCCTTGACACACACGCGCTTGTCAAAACAT
TTAACTTAAAATTAGGCATATCGATTTATGCCTTAAAGCGTACTTAAGTCTAATCTTAACCCACTTGCGCACATCTACAC
GACATCACCATAATAGTCTTATCTTGTTAATTCAAATTTTTAATGTTAACACTTCGACGGTCGCGCACACCACAGAACAC
TAAGAACATCGTAGGCCGCTGGGGATGAATGTTTGTCGCCTTTTCGGGGTTCGTCAGCAGCCTGTTCGATTAACCTGAG
GAAAGATAAAACATTTGGTTTAGAGACAACAGATCTAGATGTAGCGATTGTGCATCTAGAAGGAACTCACTTGTGGGA
TTTTTTCTCCTGCCAGGACCGCAATCAGTATTTGACCGCAATCAGTAGTGCTGTCGGTTCGGTCCTGTTACTGCTGCAGC
TATTGTGAGGCAGAGGATAATGATCCGAAGGGTTGAATTCCTGCCCCGAACGTTTTGAACGAGCGCGCTCGACACCGTC
TGGCGGCGCAATCGGAGCACCCGGTGACGTCTGGGCCTCTAGCGTGTTGTGCTGGTAGAGCCCGACTCGTCGCCGTACA
GCTTCCGGGGGATCTTGTTGAACGGTTCCTTGTAACACCACGTTCCAGAATAGAGGAAAATTGGCTGCAAAAAAAAAA
GGAATTAACACCATGATTAGCATCCTAGTCGCTAGCTTCGCCAAACAATCTTCTTCTTCTTTCGTGGCCCACTTACAACT
TACCGAATCATACACAATCATACACAGCGCACAGTCGCAAACACCTGAGGCAGGCCCATATAATTTCTGCATTATATCT
GCAAGTTAAAAGAGGCCATAGTCTATTAAAATAATGTTTCTTTATCACTATAATCGCTGGATAACGCTTACCCACTGTCT
TGTGCTGTGTTGTCCACCACACCACTTTTCTATGCCGGTACATGCACGTACCGCTTCCGACGGAGCCAGCACGCATTGG
GAAGGACTTCTACCACTTACTTTTGCTAAAATGGCACTTACTTACGTAAAATTAAGAACTTAATTATTAAAAAACTTACT
AAAATTGATCAGCCAACAACACTGTTTACGTTTTTTTACACACACAACTTGACTTCTACAGTGACAGCAGCCGCATAGC
ACCAACACAACGAAAGGTGTCAGACGATCTTAGGCGAGGGGTAGGAAGGAGATCGGTGGTGAGGGACGTGAAAGCTC
AGCTCGACAGAATCGCTATAGGAGGAAGCGAGGCAACTAGACTAGACTAGAGAAATTAGAGCGAGAGGTACCTCACC
AACCCTCGCATTGTTTGCCGGGATATCATTGACCGAAAGAAAACAGTTGATAAACGATGGTAAAGGTAAGGCTGTATA
AGCGATAATAGAAAATATGTTTGCATCAAAATAGCTTCAAATTTTATTGCATTCAGTTGAAATCATAGTCCAACCTATC
CGAACTTTTTTTTTATTTTCCTTTTTATTAACCCGAGCACAAACTGCATACTTGTAGTTGTTGGTACAGTCTTAGGCGGCG
CTCTGGCGCCAATCGAACAAAACAAACAACCACCACTCTGTTCGAATGGCGCTCTGTTTCGATGTCTTATAGAACTGTC
ATTATGCACTGCAATACGCCTATCATTCCATCATGAGAAGCATGAAGCATTTTCATAATTTTAATGAATATACATCTACA
GGGGGGAGATTCAAAAATAATTTACTAGTAATTCACATCAATAAAAACATAAAAATGATTTCAATTTCATATTGCAAAA
TGTAAACAAACGTCCATGCCAAAACACATACAGTGTATACACATGCAGAATACGTAACCACGTAACCATTGATAATCG
ACTATCGCAAGGAATTTTACGACTGATACACGAAGGGGTGATGACGAATGCGGCCCTAGCTGCAGCCAGACGTTTGCG
TTTGTCGCAAAAAGTTCTGCGATTGTCGCAAAAACTTTTGCGATTGTCGCAAAAACTTCTGCGATTGTCGCAAAAACTT
CCGCGATTGTCGCAAAAACTTCTGCGATTGTCGCAAAAACTTCTGCGATTGTCGCAAAAACTTTTCGGTAAAAGTAAGG
CGGCGCTCTAGCAGCAATCGAACACGACATCCGACACGAACAAACCCATATAATCATATGGAGGTGCACTGTCAGACA
CAAACAAACAAACAAGACAAACGTCGATTCGAACATGTCAGTTGATTCTGTCTGTGATGTTCGATACCCACCTGTGCTG
TGAGTACCTTGGCTGTCAAACGCTTCACAGCTGCAGTAGATAGAATTCAATTCGGGACATTTTTAAGTTTGTTTACGTAG
TTTGTCTCTTTTTCTTCTTAAAATTGCGTGCAGAATATTTAAAATAGCTATCAATAATTATTGTTAAACTTTTCAATCAAT
TATAACATCAAATATAAAGGATTGCAACGTATTAAACTATTGTGTGTACATAATCCATGTGTATGCACTGTATGTGTTTT
GGCATGGACGTTTGTTTACTTTTTTCAATATTAAATTTGAATGATTTCTATGTTATTATAGATGTGAATAACTAGTAAAT
TATGAGTTGAATCTTCCCCCTTTAGGTGGATATTCATTTAAACTATGAAAATGCTTCATTTTCGAGACGAAAAGAAAGG
CGTATTGCAGTGCATCATGACAGGTCTATAAGACATCGAACAGCTGACAGAGCACCTATCGAACAGAGTCGTGTTTGTT
TGTCTCGTTCAAGGTGTTATAAATGACAAGGTGAAGTTGTTCAGAGCAAAATGACATTTCTCGACTTGACATTTCTCTTC
CGGGGGCTCTGGTATAAAAATCGGGGAGGACTGGTGCGGGGTAATGAAATTTGTATGCAGAGAGTGACAGATGTCCCC
CACAATGTCGCCCAGCAAAAGCGATAAAAATCAACCTTCTAAATACATTATCCTTGGTCTCG

These trace mate-pairs (from the S WGS) cross the breakpoint on the telomeric end of the b inversion (11C - outside inversion; to 12E -inside inversion)on PEST)

>gnl|ti|1429590608 name:1101671911437 mate:1429578773
GGGACGCGTCAGAGAGAGATGAGAGAGAAGAAGAAGAAGAAGAAGAAGAAGAAGAAGAAGAAGAAGA
AGAAGAAGAAGATGAAGAAGATGAAGAAGATGATGATGAAGAAGAAGAAGAAGAAGAAGAAGAAGAA
GAAGAAGAGGAATTGGAAGAGGAAGAAGAGAAATTGGAAGAGGAATTGGAAGAGGAATTAGAAGAGGAAG
AAGAACAGTTTGTGAATCGGTAAACTATTTTAAAATTGGTAGATTAATCCGTTGGTCTGATCTCATAAAA
AGCAAACCAATATTAACAACCTATCATTTGACCGTGGAAAGCGATTCACACAAACAGTTTAGAATAGACC
ATTTCTTATTTTTTCTCTAAATTTGCGAATGTAGTAACGTTTTTCTTTTCGTTAATTCAAAATTATCATG
AATGATTGCACTCATTTTCATGATTTTTTTTTATTTTGGTGCGTCAAAAGAACCAAGCGAACCATTTTAT
TCATTTTTTAACGTGAACTGATTGAACCGTACAGGTCTGGATCATTTGGCATACCTATAATTTGCACATG
AGCTCTATTTACAAACAAAAACATTCGAACACGAGGCAAACAACCGAAACGCCACAATCAACCTACAATC
ACTCGCATGGCTTGCGTGTACCATTTTTGAATGTGGTTCACTAACTACATCCCGCACTTGCTCCAATACT
CTCTTTTAGCAGCGAATATTGCTTTGCTCAATCACTATTTTCCCCATTGACCGACAATCGAGCCCCGAAA
GCGGAACAGTACATTTCCTGTTTTTGTACCGTTGTTAGATTTTGATTAAAAAAAATGCATCAATTGAACG
TATCATATTACTTGGAGGAAGATCCACCAACAGCTTGTAGAATATCCTGT

>gnl|ti|1429578773 name:1101555818176 mate:1429590608
TGTTCTGGACGGGGAGCGAGGAGGAGAGGAGGAGGAGGGAGGGGACGACGGGGCCCGCGCCCTGAAGGTG
GGATCGTCCACGGCGGCTGGCAGCACGGCGCTGGAAGCCTGCTGCCGGGAGGATGCCCTGTTCCCCTCGA
CCACGCTCGGCCGGCGGCGCTGCCGGCGGGGCCACTCGATCAGACGGTCCCGGTTGCGGAACTCGTTCAA
GACGCCCAAGGACGTGGAGGCGCGGGCACCGCGCAAGAACGAAAACATCGTGCACATCAGCCCGATGAAC
AAGTCGGTCGACTCCATCCTGGACACCATTAGCCAGGAGGAGGGTGAAGAAGAGGAGCAGGAGGAGGAGG
AGCGGATGAACGATGGCGAGACGGCACCGGAGACGGGTGGCTCGCAGCCGCCGGAACCGTCAGCCAAGCT
GTGCAAAGCGTCCAAGCTGTCGCACCACTCCGACTCGTCCGAGGATGACTTCGAAGCGTTCATGGCGCAC
TACAACTACAACTCGAGCGAACCGCTCTCGACGGAACTGATCTCGTACTTCGACATGAAGCTTAACCCCA
CCGCCGTGTGCAGCATGCACGATCTGAGCCAGCACCATCACGGCAGCCTGCCGGTAACGCTCGAGGACAC
CCAGCGGCTAGCGCTCAGCCTGGACGATCTGGACCACTACTCGCTGCCGGCCGGGGTTGGCGAGGCGGAC
GACGCTGCGCTGGCCGGGCTGCGCACCCGCGAGCTGAAGGACCGCGAGGACGAGCTGGACCGGGACGAGG
TGTTCTTCAACCAGGACGAGGTGCTGGAGATGCTGCGCACGGCGGCAGAGTCGGCCGCGTCCGCTTCCAC
CGCCCAGCGCCGGCTATCCGACGACCGGCTGCTGCGCCATTCCGGCTGCCTGCACGGGGAGGACGATACG
GCCCCCCGTCGCTGCTGTGCGTGACG

>gnl|ti|1429585827 name:1101671815450 mate:1429585443
GTAGCGGGAGTCGAGAGTACTTACTTACTTACTTACTTGTTTGGAATGGTAATATTTAGAAATTCAAGTT
TCCGATGCATTTTACACACAATTCAATAATAGATTAAATGTTTTATAATAGTTAATAGTTCCATTGAAAA
TGGAACAAAGATGAGCCATATTCGGAAAAAAGAAAATCAGTCTAGAAAAGTTTTATCAAATAACTGCATT
ACAGAATGAAACGGAAACGCGTATACTTGAGATCTATTCTCTTTGTCTCTTTATTACTTAATAATTTTCG
AAATAGGGAGTAATATGAGAAGAAGAAGAAAAAGTAAAGGAATAAGAAGAAGAAGAAGAAGAAGAAGAAG
AATAAGAAGAAGAAGATGAAGAAGAAGAAGATGAAGAAGAAGAAGAAGAAGAAGAAGAAGAAGAAGAAGA
AGAAGAAGAAGAAGAAGAAGAAGAAGATGAAGAAGATGAAGAAGATGATGATGAAGAAGAAGAAGAAGAA
GAAGAAGAAGAAGAAGAAGAAGAGGAATTGGAAGAGGAAGAAGAGAAATTGGAAGAGGAATTGGAAGAGG
AATTAGAAGAGGAAGAAGAACAGTTTGTGAATCGGTAAACTATTTTAAAATTGGTAGATTAATCCGTTGG
TCTGATCTCATAAAAGCAACCAATATTAACAACCTATCATTTGACCGTGGAAGCGATTCACACAACAGTT
TAGAATAGACCATTTCTTATTTTTCTCTAATTGCGAATGTAGTAACGTTTTCTTTTCGTTAATTCAAATT
ATCATGAATGATTGCACTCATTTCATGATTTTTTTTATTTGGTGCGTCAAAGACAAGCGACATTTATTCA
TTTTTACGTGACTGATGACGTACAGTCTGGATCATTTGGCATACTATATTGCATGAGCCATTACACAACA
TTCGACCGAGCAACACGAACGCCATCACTACATCTCCATGCTGGTACATTGATGGTCCTACTATCGCTGC
TCATCTCTTAGCCGATGCTGCCATC

>gnl|ti|1429585443 name:1101671811226 mate:1429585827
TTAACGTTGAGCTCGTTTTGTTAGCCTTAACTTTGCTACATTGTTGTGCAGTGTGACGAAGAAGCAGAGC
TGTGTCCGATTGAGGACATGATAGGTGGGAGTGCAAATGTGCAAACGGGGAGAGGGTGTAGTACACTCCC
CGCAGCTCATTTGCTTGCTGCTTTGTTGTTAAACATTTAGCTTTACACACAAGGGTTAGCCGATTAGCTT
AGCCGTTAGTGTAGTTGCGAGTTGCGAACGTAACCGCGCCTGAGCGATTTGGTTTCCCAGGTTTCCAAAT
CGCCCGGTCAAAAGAACGGGACGATTCAATCAGATACCAAATAACTTATTTTATAATCAACAATAACACA
TAGCAAACACCTACAGAGAGCACAGGATTACGCTTAGTCCTTATGTGCGCAACAATGCAACTCCAGGCTG
ACAATGCGGGTAGGTTTTCGTGCACTAATCGAATGCAGAGAAGCATCCAATGCCTAAATGATGATATTAT
CGATAATCACACGCATCCCCAATTTATAAACGAGACCTGCGACGTTGCTAAGAACAGCATGGTTATCAAC
TCCTCCGAACATGAATCTAATCAGTTCCCCCACCAGTTGAGGATTGTTGGGCCCATGTGCCGCTACGCGC
TAGAAAAATCTAGTGTCTAGACTGCATCCACTGCTACGCACCATTGGCAGTAACCGCCCGTTGGTAGATG
GTGGTTACTACCACGGCACGGCTCTGAACATAATCTAAAACAGCGCATAATGGGGAACACTTGGGAACAC
TTACAACAGATAGTTAACCGCGGGAGCAAAGATAAGTAGCAGTAGTAGTGATAGCAGTAGCAGTAGTGAT
AGTAATATTGTACTAAAAATAGACATCTACATGTGAGGACGTCGATGTGACACGTGATGGACATTGTACA
GGGTGGGCTTGTGCACACCGGGTGCTGCTAATGGAGCTGGAAACCAAGACATAGACTATATACATAGCAG
TGGATGTGTTATCTTTCTCTCGCTCTCTCTGCTCACTACCCGCTACGGAGGGGAGGGGTGTATCATGGTA
GGACAGGGCAGGAGAG

These trace mate-pairs (from the S WGS) have one mate-pair outside the breakpoint on the centromeric end of the b inversion (11A - outside inversion) and the other end in the repeat sequence at the inversion breakpoint.

>gnl|ti|1441840352 name:1101854097862 mate:1442018046
TGAGGTGGTAGCGATAGTCTTTCCGACACAGTCCACAGTAAGGTGTTCCAGGGAGCAGAATACACAAGCG
GTTTTATGTTGGGCATGTGCTTGTGCGATGCTTAGAAACACAGAGAGAAATAACGATACTATAATCATCA
GACAAGAGTTCGTGAATGTCTCCTAACAGATCTCAAAGCTACTGAAATTTGGCCATTTGCCATCTAGTCT
ATTTACTTTAATATAACCTATTTACAAATCGCCACGGGCTGGACCTACAATTTGTTTTATACTTTTCTAA
TAGTAGATCAGCATATTGTATTATTTTTATCATATTTGTTCTTTGATGAATGTCACTAGTACTATGCCAA
AGTGGTAAATTAAGGATTCGTTTAAGAAATTTGTTTTGGAACACTTGGATTTTCTTTAAGTGCGTAGAGG
CACTTTTTTCCCAAACTGGGTATGCATATAATAATATAGGTCGTAGGATTTGTTTATAAAGCAAAAGCTT
AGTCAAAATACCTATTTGTTTCAGGTATTTTATTTAAGAATCATCAAAGTCTGAAATCGTTACACAAACA
AACAACACAAGACCGCGGTACTCTTTCACAAATCGTCGTGAGTCAATCGCAAGACTGTTAACGATACAGG
GTCGGCGATTGTCGCTAAAAATCTTCGGTGATTGTCGCAAAAAGTTCTGCGATTGCCGCAAAAACTTCTG
CGATTGTCGCAAAAAGTTCTGCGATTGTCGCAAAAAGTTCTGCGATTGTCGCAAAAAGTTCTGCGATTGT
CGCAAAAGTTCTACGATTGTCGCAAAAAGTTCTGCGATTGTCGCAAAAATTCTGCGATTGTCGCAAAAAT
TCTGCGATTGTCGCAAAAGTTCTGCGATTATCGCAAAACTTCTGCGATGTCGCAAAACTTCTGCGATGTC
GCAAAACTTCTGCGATGTCGCAAAACTTTGCGATGTCGCAAAACGATGTCGCAAAACTTTGCGATTGTCG
C

>gnl|ti|1442018046 name:1101854183687 mate:1441840352
GTGGGGTGAGTCGCAAGTCGCAGAGTTTTTGCGACAATCGCAAAAGTCGCAGAAGTTTTTGCGACAATCG
CAAAAGTCGCAAAAGTTTCTGCGACAATCGCAAAAGTCGCAGAAGTTTTTGCGACAATCGCAAAAGTCGC
AAAAGTTTTTGCGACAATCGCAAAAGTCGCAGAAGTTTTTGCGACAATCGCAAAAGTTTTTGCGACAATC
GCAAAAGTTTTTGTGACAATCGCAAAAGTTTTTGCGACAATCGCAAAAGTCGCAGAAATTTTTGCGACAA
TCGCAAAAGTCGCAAAATTTTTTGCGACAATCGCAAAAGTCGCAGAAGTTTTTGCGACAATCGCAAAAGT
TTTTGCGACAATCGCAAAAGTCGCAGAAGTTTTTGCGACAATCGCAAAACTTTTTGCGACAATCGCAAAA
GTCGCAGAAATTTTTGTGACAATCGCAAAAGTCGCAAAAGTTTCTGCGACAATCGCAAAAGTCGCAGAAG
TTTTTGTGACAATCGCAAAAGTCGCAAAAGTTTTTGCGACAATCGCAAAAGTCACAGAAGTTTTTGCGAC
AATCGCAAAAGTCGCAGAAGTTTTTGCGACAATCGCAAAAGTCGCTAAAGTTTTTGCGACAATCGCAAAA
GTCGCAAAAGTTTTTGCGACAATCGCAAAAGTCGCAGAAGTTTTTGCGACAATCGCAAAAGTCGCAAAAT
TTTTTGCGACAATCGCAAAAGTCGCAGAAGTTTTTGCGACAATCGCAAAAGTCGCAAAAGTTTTTGCGAC
AATCGCAAAAGTCGCAGAAGTTTTTGCGACAATCGCAAAAGTCGCAGAAGTTTTTGCGACAATCGCAAAG
TCGCAGAAGTTTTTGTGACATCGCAAAGTCGCAAAATTTTGCGACATCGCAAAGTCGCAGAGTTTTGCGA
CATCGCAAAGTTTTGCGACATCGCAAAGTCGCAGAAGTTTTGAGCAGTTGCTATACGACATAGTAAAGTA

>gnl|ti|1429500427 name:1101671247099 mate:1429497417
TGACGTCTGAGGGTGCGCTTTCGACTGCTGCCGATCGAAGTTGAACGCACCGACGTTGAGTGTTTTCCGG
GGACTTTGCTCGGTGGCCGGCACCGCCACACGCTCATTTGCCGTCGATTCACGCTCCCCTTCGCTGCCGT
AGCGCGAGTAGAAGTTGCGCATAATGTTCGGAACGATGGGATCCGCTTCCGGCGCTATAGACGGTGTGGC
AAGCGCCGACACGGCCATGCTCGTAGATCGTAGCAGCTGCTTGCGACGTTGCGTTGCCTGCTCGGCGGTG
CTCCACGGATAGCAGCGGCCAACGATCTTCTGGCTTTCGAAGCGCTGATAGCCGGTAATGATGGTGGCCA
TCGTGAGGAAGGAGCAGACGACGCTGATCACCTGCACCGTGACCGTGTCGTAGTTGCGGAACGTGCCGTC
CCGCAGCAGGATGTACAGCTGCAGCATCATCTGGGGGGCGGCCTGCAGGAACGCCTGCAGAAAGTGGTAC
AGCTCGCAGGGAGACGTTTCCGTTATCTTGGCGACGGCTTGCGCACGGCCGTAGGAGCCTTTGTCGTGGA
ACAGGGCTTCGACGCTCCAGAAAATGCGTCGGCTGAAGCTGAAGGGAGAGAGCGAGAGAGAGCGAATGAG
TGCGTTTGACGATGTGAATCATTGCGCTGAAGGGGGGGGGGACTTACCGGTAGAGAGCGGCGAGCGGAAA
GAACACAAGTATCGCGACTTGGTTGAACAGGAAGCGACCCGTACGCTCGTCAAATCCAATGCGTTCCGGC
CACCGTTCCGGGGTGGATACGATGGAGCAGAAGCACACCACGGCCGGGAGCCAGATAAGGGTGAGCGTGA
ACCATGCCCACGTCGGAGCGCCGTTGCGGAAGTGCTCCACCGTGATCAGTATGTCCGCCGTCGTCAGCAC
AATGTACAGCATCAGCTCGAAGATCACCGGCACCAGGAAGTGGCCACTATTTGGGTGCGCGTCTGACCGG
GTAGCGAGCACGATCGTGCACACATCGAGGGCGTCATCGTGCAAACTCCTCTTTGCGTTACTGGCTCCCC
AAACGATTGATGTAAGTCTAGCCGCGCCACCAGTGT

>gnl|ti|1429497417 name:1101671153211 mate:1429500427
TTAGCCTGGCAGCGAATCGTAAGTCGCAGAAGTTTTTGCGACAATCGCAAAAGTCGCAAAAGTTTTTGCG
ACAATCGCAAAAGTCGCAGAAGTTTTTGCGACAATCGCAAAAGTCGCAAAAGTCGCAGAAGTTTTTGCGA
CAATCGCAAAAGTCGCAAAAGTTTTTGCGACAATCGCAAAAGTCGCAAAAGTCGCAAAAGTTTTTGCGAC
AATCGCAAAAGTCGCAAAAGTTTTTGCGACAATCGCAAAAGTCGCAAAAGTTTTTGTGACAATCGCAAAA
GTCGCAAAAGTCGCAGAAGTTTTTGCGACAATCGCAAAAGTCGCAAAAGTTTTTGCGGCAATCGCAAAAG
TCGCAAAAGTTTTTGCGACAATCGCAAAAGTCGCAGAAGTTTTTGCGACAATCGCAAAAGTCGCAAAAGT
TTTTGCGACAATCGCAAAAGTCGCAAAAGTTTTTGCGACAATCGCAAAAGTTTTTGCGACAATCGCAAAA
GTCGCAAAAGTTGCAAAAGTTTTTGCGACAATCGCAAAAGTCGCAGAAGTTTTTGCGACAATCGCAAAAG
TCGCAGAAGTTTTTGCGACAACCGCAAAAGTCGCAGAAGTTTTTGCGACAATCGCAAAAGTCGCAAAAGT
TTTTGCGACAATCGCAAAAGTCGCAAAAGTTGTTGCGACAATCGCAAAAGTCGCAAAAGTCGCAAAAGTC
GCAGAAGTTGTTGCGACAATCGCAAAAGTCGCAGAAGTTTTTGCGACAATCGCAAAAGTCGCAGAAGTTT
TTGCGACAATCGCAAAAGTCGCAGAAGTTTTTGCGACAATCGCAAAAGTCGCAGAAGTTTCTGCGACAAT
CGCAAAAGTCGCAGAAGTTGCTGCGACAATCGCAAAAGTCGCAAAAGTTGTTGCGACAATCGCAATAGTC
GCAAAAGTTGTTGCGTTATCGAATAGTCGCAAAGTTGTAGCGCATCGCAAAGTCGCAAAAGTTTAGCGAC

ATCGAAAGTCGCAAAAGTCGCAAAGTGCAAAATTTTGCGACATCGCGAAGTCGCAAAGTTCTGCAGACAT
TGCAAAGTCGCAAAGTTTTGCAATAATCGCCATAGTCGCAATAGTCGCTAGAGTC

>gnl|ti|1430050374 name:1101693882874 mate:1430031143
TTACGGCTGAGATAGAGTTGCGCATAATGTTCGGCACGATGGGATCCGCTTCCGGCGCTATAGACGGTGT
GGCAAGCGCCGACACGGCCATGCTCGTAGATCGTAGCAGCTGCTTGCGACGTTGCGTTGCCTGCTCGGCG
GTGCTCCACGGATAGCAGCGGCCAACGATCTTCTGGCTTTCGAAGCGCTGATAGCCGGTAATGATGGTGG
CCATCGTGAGGAAGGAGCAGACGACGCTGATCACCTGCACCGTGACCGTGTCGTAGTTGCGGAACGTGCC
GTCCCGCAGCAGGATGTACAGCTGCAGCATCATCTGGGGGGCGGCCTGCAGGAATGCCTGCAGAAAGTGG
TACAGCTCGCAGGGAGACGTTTCCGTTATCTTGGCGACGGCTTGCGCACGGCCGTAGGAGCCTTTGTCGT
GGAACAGGGCTTCGACGCTCCAGAAAATGCGTCGGCTGAAGCTGAAGGGAGAGAGCGAGAGAGAGCGAAT
GAGTGCGTTTGACGATGTGAATCATTGCGCTGAAGGGGGGGGGGGGACTTACCGGTAGAGAGCGGCGAGC
GGAAAGAACACAAGTATCACGACCTGGTTGAACAGGAAGCGACCCGTACGCTCGTCAAATCCAATGCGTT
CCGGCCACCGTTCCGGGGTGGATACGATGGAGCAGAAGCACACCACGGCCGGGAGCCAGATAAGGGTGAG
CGTGAACCATGCCCACGTCGGAGCGCCGTTGCGGAAGTGCTCCACCGTGATCAGTATGTCCGCCGTCGTC
AGCGCAATGTACAGCATCATCTCGAAGATCACCGGCACCACGAACGTGACCAGTATTTAGGTGCGCGTCT
TGACAGGGTAGCCGATCACGGATGGTGGTTCACGGTCGAGGGCGTCTATCGTGCAAACTCTCTATTGCGG
TAACTGGCTCACAAACGATAGATGTTTGTCTAGCGCTGCAATGAGGTGTGAGTATGAGTGGATTGCGATC
TATACAGTCGTCTGCATCGTATCGGTAAGACTATACCGACCAATCTGACTCATGGGCTGCACATGATTAG
TCACCATGGCCCGTACAATCATTCGTCTGATGCAC

>gnl|ti|1430031143 name:1101692130481 mate:1430050374
TTAAAGGGAAGCACGTTTTTGCGACATCGCAAAAGTCGCAAAAGTTTTTGCGACAATCGCAAAAGTCGCA
AAAGTTTTTGCGACAATCGCAAAAGTCGCAAAAGTTTTTGCGACAATCGCAAAAGTTTTTGCGACAATCG
CAAAAGTCACAAAAGTTTTTGCGACAATCGCAAAAGTCGCAGAAGTTTTTGCGACAATCGCAAAAGTCGC
AAAAGTTTTTGCGACAATCGCAAAAGTCGCAGAAGTTTTTGCGACAATCGCAAAAGTCGCAGAAGTTTTT
GCGACAATCGCAAAAGTCGCAAAAGTTTTTGCGACAATCGCAAAAGTCGCAGAAGTTTTTGCGACAATCG
CAAAAGTCGCAGAAGTTTTTGCGACAATCGCAAAAGTTGCAAAAGTTTTTGCGACAATCGCAAAAGTCGC
AAAAGATTTTGCGACAATCGCAAAAGTCGCAGAAGTTTTTGCGACTATCGCGGAAGTCGCAAAAATTTTT
GAAACAATCGCAAAAGTCGCAGGAAATTTTGCGACAATCTCAAAAGTCGCAGAAGTTTTTGCGACAATCG
CAGAAGTGGCAAAAGATTTTGCGACAATAGCAAAAGTCGCAGAAGTTGTTGCGACAATCGCAAAAGTCGC
AAAAGTTTTTGCGACAATCGCAAAAGTCTCAAAAGATGGAAAAGATTTTGCGACAATCGCAGGGTCGCAG
AAGTTTTTGCGACTATCTCAACAGGAGTCCAAGCAGACAGGGTTTTGCGACAATCCCGAAAGTCCCAAAA
ATATTTGTGACAATCTCAGAGTTTCAAAAGTGTTGCCCAATCGCAAATGCCCACAATGTTTGCGACTATC
GCCAAGTTTCAAAGCTATGCGACATCAAGAAGTCGCAAAAATTTGCCATATGTAAAGACGCAAAAAGTTG
CGAAACGCAAAGCCCATAATA
